# Perception and Performance on a Virtual Reality Cognitive Stimulation for Use in the Intensive Care Unit: A Non-randomized Trial in Critically Ill Patients

**DOI:** 10.3389/fmed.2019.00287

**Published:** 2019-12-10

**Authors:** Stephan M. Gerber, Marie-Madlen Jeitziner, Samuel E. J. Knobel, Urs P. Mosimann, René M. Müri, Stephan M. Jakob, Tobias Nef

**Affiliations:** ^1^Gerontechnology and Rehabilitation Group, University of Bern, Bern, Switzerland; ^2^Department of Intensive Care Medicine, University Hospital Bern (Inselspital), University of Bern, Bern, Switzerland; ^3^Department of Neurology, University Neurorehabilitation, University Hospital Bern (Inselspital), University of Bern, Bern, Switzerland; ^4^ARTORG Center for Biomedical Engineering Research, University of Bern, Bern, Switzerland

**Keywords:** virtual reality, critical illness, intensive care unit, cognitive dysfunction, nature, stimulation

## Abstract

**Background:** Newly acquired long-term cognitive impairments are common among survivors of critical illness. They have been linked to the stressful situation that patients experience in the intensive care unit (ICU). In this paper we use virtual reality (VR) technology to comfort critically ill patients and reduce stress during their ICU stay. We investigate the acceptance, comfort, recollection, and visual perception of VR stimulation and how it affects physiological parameters.

**Methods:** A VR head-mounted display was used to present immersive nature scenes to 33 critically ill cardiac surgery patients [mean age 63 years (range 32–83)]. Data was collected with an eye tracker fitted inside the VR head-mounted display to measure eye movements (250 Hz) and sensors to record physiological parameters (240 Hz). Patients received VR stimulation (for 5 min.) prior to ICU admission, during ICU stay, and 3 months after discharge. Acceptance, recollection and comfort were assessed with validated questionnaires.

**Results:** The number of gazed meaningful objects per minute was significantly lower during the ICU session compared to pre- and follow-up sessions, whereas mean duration of fixation on meaningful moving objects did not differ between the sessions. While respiratory rate decreased significantly during VR stimulation, heart rate and blood pressure remained constant. Post-ICU rating of VR acceptance during ICU stay was moderate to high and discomfort low. Recollection of VR was high [28/33 patients (84.8%)], while recollection of ICU stay was low [10/33 patients (30.3%)].

**Conclusion:** Eye movements indicate that patients were able to perceive and process cognitive stimulation during their ICU stay. VR was recalled better than the rest of the ICU stay and well accepted. Decreased respiratory rate during stimulation indicate a relaxing effect of VR.

## Introduction

The intensive care unit (ICU) is a noisy and stressful environment in which critically ill patients are exposed to psychological stress factors, including sensory overload and deprivation, isolation, temporal disorientation and a feeling of lack of control (1, 2). After ICU stay, ~50–75% of all critically ill patients will suffer from newly acquired long-term cognitive impairment (3–5). These cognitive long-term effects were linked to the stressful situation that patients experience during their ICU stay (4, 6). They negatively affect health-related quality of life after ICU discharge and reduce the effectiveness of intensive care (7–9).

We therefore embarked to develop a novel strategy using virtual reality (VR)-based cognitive stimulation technology to comfort critically ill patients during their stay in the ICU. When wearing a head-mounted VR display, patients do not hear or see the ICU and they are virtually “taken out” of the ICU and moved into a pleasant virtual environment. The virtual environment can be designed to the needs of immobile critically ill patients and applied directly in bed.

We decided to present pleasant nature scenes to critically ill patients because others have shown the beneficial effects of exposure to nature scenes. A study with 38 healthy participants described the cognitive benefits (i.e., directed attention) arising from the interaction with nature (10). In another study, Gamble et al. found that looking at nature pictures improves executive attention in older adults (11). There are two complementary theories related to the restorative effects of natural environments i.e., positive emotional state, reduced physiological activity level and increased cognitive functions. The first theory, called the Attention Restoration Theory, states that directed (i.e., high-effort) and voluntary (i.e., low-effort) attention, which influences cognitive performance, is a limited resource and can be depleted over a prolonged period of time. Directed and voluntary attention can be restored by interacting with nature environments (11–13). In rather stressful environments like the ICU, however, one must be vigilant, causing cognitive functions to decline. The second theory, the Stress Recovery Theory, postulates that nature environments reduce fear and anger, and have a stimulating effect on the parasympathetic nervous system shown in physical markers of stress (e.g., decreasing respiratory rate) (14). An established line of research suggests that exploring nature in VR has a restorative effect, similar to being outside in nature (13).

The state-of-the-art therapy provided to critically ill patients focuses on physical rehabilitation, and there is currently no consensus on how to prevent cognitive impairments after ICU discharge (15, 16). However, Turon et al. showed that training of cognitive functions presented on a television screen is feasible and may be beneficial for critically ill patients (17).

In a previous study, we demonstrated that healthy participants stimulated by nature videos through immersive VR in the ICU were more relaxed after the stimulation. Additionally, visual attention was not depleted during the course of the VR stimulation (18). Our finding suggests that participants were highly immersed and able to ignore the actual surroundings and restore their visual attention. To our knowledge, it remains unclear whether VR stimulation in critically ill patients would produce physiological and psychological restorative effects comparable with those observed in healthy participants.

The aim of this feasibility study in critically ill patients was to investigate the acceptance, comfort, recollection, visual perception and processing of immersive nature-related VR stimulation, and how VR affects physiological parameters. We hypothesized the following: firstly, that VR stimulation using the new proposed method offers high usability, immersion and satisfaction, is recollected and does not promote discomfort; secondly, that patients would be able to perceive and process the VR stimulation (i.e., visual attention) before and during their stay in the ICU; thirdly, that VR stimulation will reduce physiological markers of stress.

## Materials and Methods

### Participant Recruitment

This study was approved by the Ethics Committee of the Canton of Bern, Switzerland (KEK no. 2016-01652) and was registered at Clinicaltrails.gov (no. NCT03025373). The required sample size was calculated using the G^*^Power 3 software (19) based on a power of 80 and alpha error of 0.5. The effect size was estimated as medium-large according to the classification by Cohen, on the basis of our previous experience with VR stimulation in the ICU (relaxing effect of the VR stimulation, i.e., physiological measures), which was resulting in a sample size of 50 patients (18). Inclusion criteria were age >18 years, no neurological disorder, including delirium and planned elective open-heart surgery. Patients were recruited on admission to the ICU between 17 April 2017 and 13 March 2018.

### Procedure

The study was split into three phases (Table 1): (i) An initial session 1 day prior to ICU admission (pre-ICU session), (ii) a second session the day after surgery (ICU session), and (iii) a follow-up session ~3 months after ICU discharge (follow-up session). In the initial session, patients were briefed, signed the informed consent, filled out questionnaires on demographics, and health-related quality of life (EQ-5D-5L) (20) and cognitive functions [Montreal-Cognitive-Assessment (MoCA) (21)] were assessed. Patients were then prepared for the VR stimulation by a study nurse, i.e., lying on the bed and monitoring of physiological parameters. After 10 min of rest, patients were stimulated with the nature VR environment for 5 min. Patients were stimulated in the ICU the morning after surgery the second time. Sedation level RASS, GCS, and delirium [Confusion Assessment Method for the ICU, CAM-ICU (22)] were assessed by trained ICU nurses. In the follow-up sessions, patients were stimulated again and questioned about their memories of the intensive care experience and acceptance, comfort and memories of the VR stimulation. The assessment of cognitive functions compared to the registered clinical trial a deviation exists. Since the Consortium to Establish a Registry for Alzheimer's Disease (CERAD) (23) was too time consuming for patients and had a too high workload instead the MoCA was used. There is evidence that the MoCA assess cognitive functions at a comparable level but is much shorter in time and needs less workload (24).

**Table 1:** Study procedure.

**Tasks/variables**	**Pre-ICU session**	**ICU session**	**Follow-up session**
Time since inclusion	Day 1	Day 3	Day 90 (95% CI 80.10)
Informed consent	X		
Demographic questionnaire	X		
VR stimulation	X	X	X
Monitoring of physiological parameters	X	X	X
Sedation level (RASS)	X	X	X
Consciousness (GCS)	X	X	X
Delirium (CAM-ICU)		X	
Cognitive functions (MOCA)	X		X
Health-related quality of life (EQ-5D-5L)	X		X
Questionnaire on acceptance, discomfort and recollection			X

*ICU, Intensive Care Unit; VR, Virtual reality; RASS, Richmond Agitation-Sedation Scale; GCS, Glasgow Coma Scale; CAM-ICU, Confusion Assessment Method for the Intensive Care Unit; MOCA, Montreal-Cognitive-Assessment*.

### Cognitive VR Stimulation

All measurements were conducted in a two to four-bed ICU cubicle (Figure 2). The setup to stimulate patients consisted of several elements: (i) a computer, a head-mounted display with a built-in eye-tracker and noise canceling headphones, (ii) the equipment to measure the physiological parameters, (iii) a noise-signal monitor -positioned directly next to the patient- to register noise >50 dB. The setup was audited by the Medical Technology Department of the University Hospital of Berne for hygiene and medical eligibility requirements and was approved thereafter for use in ICU patients.

The VR stimulation consisted of a video presenting aquatic worlds and landscapes for 5 min. In addition, complementary classical music was played through noise-canceling headphones. The VR stimulation device (HTC Vive, High Tech Computer Corporation, Taoyuan, Taiwan) is a head-mounted display and was powered by a graphic card (NVIDIA GTX980, Nvidia, Santa Clara, USA). Eye movements were sampled as fixation points at a frequency of 250 Hz inside the head-mounted display by an eye tracker (SensoMotoric Instruments, Teltow, Germany). During VR stimulation sessions, eye movements were streamed to a second screen monitored by the investigators.

In all three sessions, heart and respiratory rates were continuously monitored by five-lead electrocardiography at a frequency of 240 Hz (Carescape Monitor B650, GE Healthcare, Little Chalfont, United Kingdom). In the pre-ICU and follow-up sessions, the blood pressure was measured every 2 min by a cuff-based blood pressure monitor. After surgery, blood pressure was continuously measured by an invasive intra-arterial blood pressure monitor.

In the follow-up session the questionnaire on a 5-point scale (from zero to four, Table 2) was used to assess acceptance, comfort and recollection of the VR method during the ICU session. Acceptance and, thus the sub domain immersion, was assessed by questions from the Igroup Presence Questionnaire (IPQ) (25), whereas usability and satisfaction were assessed by questions from the System Usability Scale (SUS) (26). The Simulator Sickness Questionnaire (SSQ) was used to assess the level of discomfort during VR stimulation (27), and the ICU Memory tool to assess recollection (28).

**Table 2:** Questionnaire on acceptance, discomfort, and recollection of the virtual reality stimulation during intensive care unit session.

**No**.	**Question (anchors)**	**Sub-domain**	**Domain**	**Source**
1	In the virtual world I had a sense of “being there” (Not at all—very much)	Immersion	Acceptance	IPQ
2	I thought the system was easy to use (fully disagree—fully agree)	Usability		SUS
3	I felt very confident using the system (fully disagree—fully agree)			
4	I think that I would like to use this system frequently (fully disagree—fully agree)	Satisfaction		
5	General discomfort (none—severe)	Nausea	Comfort	SSQ
6	Stomach awareness (none—Severe)			
7	Sweating (none—severe)			
8	Nausea (none—severe)			
9	Headache (none—severe)	Oculomotor problems		
10	Eye strain (None—severe)			
11	Dizziness (none—severe)	Disorientation		
12	Do you recollect being in the intensive care unit (yes—no)	Recollection of ICU	Recollection	ICU memory tool
13	Do you recollect the entire stay in the intensive care unit (yes—no)			
14	Do you recollect the VR stimulation (yes—no)	Recollection of VR stimulation		-

*ICU, Intensive Care Unit; VR, Virtual reality; IPQ, Igroup Presence Questionnaire; SSQ, Simulator Sickness Questionnaire; SUS, System Usability Scale*.

### Statistical Analysis

Analyses were performed at the individual level, whereas eye movements were measured as a collection of fixation points (i.e., gaze data), where a fixation had a minimum duration of 100 ms and dispersed around 2 degrees (29). The number of gazed moving objects outside the region of interest (e.g., dolphin) was calculated. To analyze the time effect (visual attention over time), the difference between the beginning of stimulation (first 15 s) and the last 15 s of stimulation was used and tested with one sample *t*-test against zero for significance (two-sided). The differences in eye movement (i.e., mean fixation duration, number of fixations, number of gazed meaningful moving objects and time fixated on one moving object) between the three sessions were analyzed by one-way analysis of variance (ANOVA) and Bonferroni adjustment as a *post hoc* analysis. The same analysis was used to examine health-related quality of life (i.e., EQ-5D-5L), cognitive functions (i.e., MoCA) and whether VR stimulation had a relaxing effect (decreasing physiological signs during stimulation). A one-sided *t*-test (i.e., directed hypotheses) was used based on the findings of our previous VR study (18) and the assumption that the relaxing effect always involves a reduction in physiological signs. For the questionnaire ratings, a one-sided *t*-test was used to test whether the majority was above or below the midpoint of the score scale. The data was analyzed using R (30) and Matlab18b (31).

### URL

A video of the immersive nature VR stimulation and a video including the region of interest used in the analysis can be found on our website http://www.artorg.unibe.ch/research/ger/group_members/persons/gerber_stephan/index_eng.html#pane482606.

## Results

### Demographics

**Figure 1:**
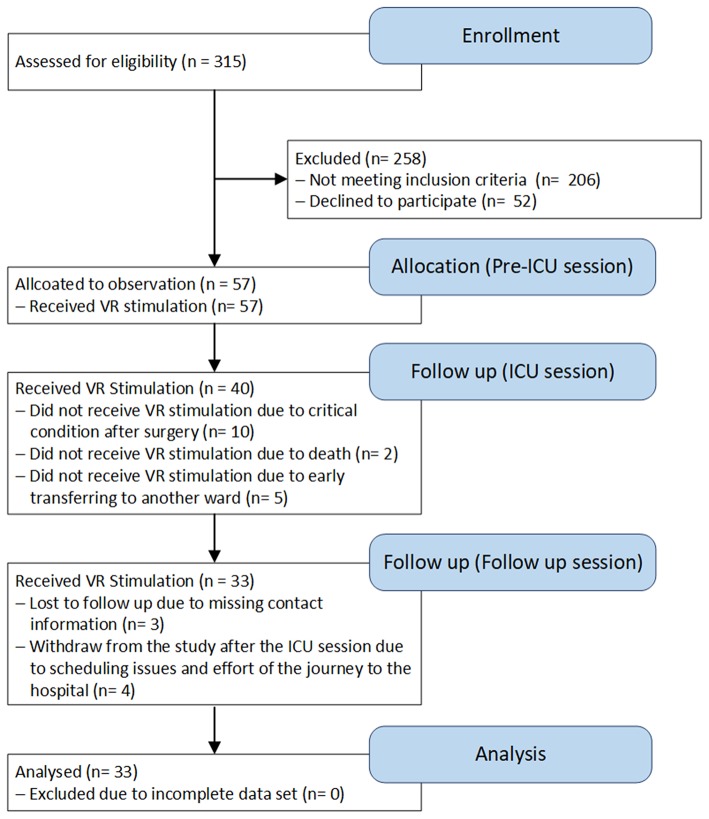
Flow of patients through the trial.

A total of 57 patients undergoing heart surgery participated in the study (Figure 1), and 33 patients (26 male) completed all the sessions (Pre-ICU, ICU, and Follow up sessions). The mean age of patients completing all the sessions was 63 years (range 32–83 years). In all the VR stimulation sessions, patients had a Glasgow Coma Score (GCS) (32) of 15 and a Richmond Agitation-Sedation Score (RASS) (33) of 0. In the second VR treatment during the ICU stay, patients had, on average, an APACHE II score of 22.3 (95% CI 20.3–24.2) and a SAPS II of 53.4 (95% CI 48.1–58.6), and all were extubated [on average within 8.1 h (95% CI 6.4–9.8)]. ICU length of stay was 0.9 days (95% CI 0.8–0.9). In the pre-ICU and follow-up sessions, 54.6 and 66.7%, respectively, of the patients received medication influencing blood pressure, while 33.3 and 69.7%, respectively, received medication influencing heart rate. During the ICU session all patients received medication influencing the heart rate, blood pressure and respiratory rate (i.e., antihypertensives or catecholamines, analgesics, sedatives). Patients who did not participate in all sessions were not considered in the final analysis and were considered as drop-outs. All dropouts were unrelated to the VR stimulation.

**Figure 2:**
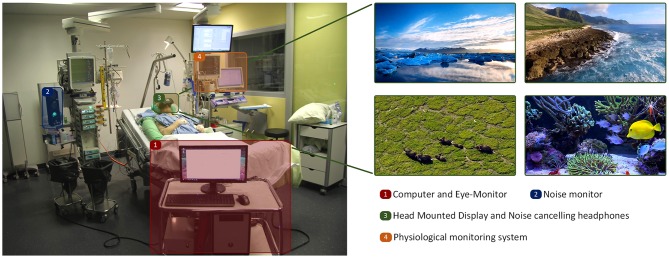
Patient during the stimulation in the ICU session, including setup and stimulation examples.

### Acceptance, Discomfort, and Recollection

**Figure 3:**
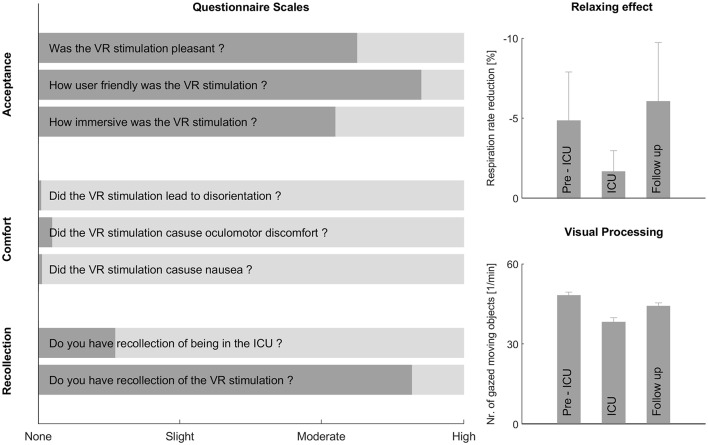
The questionnaire scales on the left show the perception of the virtual reality (VR) stimulation by critically ill patients during their Intensive Care Unit (ICU) stay. The relaxing effect and visual processing are shown on the right. Acceptance, discomfort and recollection were assessed in the follow-up session, whereas visual processing and the relaxing effect were measured in all three sessions. Overall, the new method was well accepted, did not trigger any discomfort, produced a decreasing respiratory rate during stimulation, and the patients were able to process the stimulation.

Questionnaire findings (range zero to four, Figure 3) revealed that the VR stimulation was well accepted, easy to use and appreciated by patients, as shown in the scores for high usability 3.57 (95% CI 3.3 to Inf., *t*_(29)_ = 10.78, *p* < 0.001), immersion 2.70 (95% CI 2.3 to Inf., *t*_(29)_ = 3.34, *p* < 0.001), and satisfaction 3.13 (95% CI 2.8 to Inf., *t*_(29)_ = 5.32, *p* < 0.001). Symptoms of discomfort during stimulation, such as disorientation 0.03 (95% CI -Inf. to 0.09, *t*_(29)_ = −59.00, *p* < 0.001), oculomotor problems 0.15 (95% CI -Inf. to 0.24, *t*_(29)_ = −34.00, *p* < 0.001), and nausea 0.04 (95% CI -Inf. to 0.04, *t*_(29)_ = −72.46, *p* < 0.001) were rated low on the score scale.

Based on results of the ICU Memory tool, 28 of the 33 patients (84.8%) were able to recollect the content of the stimulation (e.g., elephant, yellow fish, ocean, dolphin, polar bear, and mountains), 23 patients had a weak recollection of their stay in ICU, and 10 patients (30.3%) had a clear recollection of the whole stay in ICU. Moreover, during the VR stimulation no noise >50 dB or adverse events were registered.

### Eye Movements

Visual fixation duration was significantly higher during the ICU session compared to the pre-ICU (*p* < 0.001) and follow-up sessions (*p* < 0.001), as seen in Table 3. The number of fixations were significantly lower during the ICU session compared to pre-ICU (*p* < 0.001) and follow-up sessions (*p* < 0.001), whereas no significant difference was found between the pre-ICU and follow-up sessions. The number of gazed meaningful moving objects per minute (Figure 3) was significantly lower in the ICU session compared to the pre-ICU (*p* < 0.001) and follow-up sessions (*p* = 0.026), whereas the time fixated on one meaningful moving object did not show any significant difference. Analysis showed that the change in visual fixation duration over time within a session was not significant in any of the three sessions and remained constant.

**Table 3:** Eye movements during the three sessions.

**Variables**	**Session**	**Fixation duration (ms)**	**Number of fixations (no./min)**	**No. of gazed meaningful moving objects (no. obj./min)**	**Time fixating an object (s)**
Mean (SD)	Pre-ICU	364 (67)	595 (95)	48.1 (8.2)	0.92 (0.2)
Mean (SD)	ICU	434 (119)	351 (130)	38.1 (10.5)	0.88 (0.3)
Mean (SD)	Follow-up	372 (68)	573 (109)	44.0 (8.0)	0.97 (0.2)
ANOVA		*F*_(2, 92)_ = 6.21 *p* = **0.003**	*F*_(2, 95)_ = 46.68 *p* < **0.001**	*F*_(2, 94)_ = 10.12 *p* < **0.001**	*F*_(2, 94)_ = 1.01 *p* = 0.369
Adj. *p*-val.^*^	Pre—Fol.	1.00	1.00	0.214	
Adj. *p*-val.^*^	Pre—ICU	**<0.001**	**<0.001**	**<0.001**	
Adj. *p*-val.^*^	ICU—Fol.	**<0.001**	**<0.001**	**0.026**	

SD, Standard Deviation; Adj., Adjusted; Pre., pre-ICU session; ICU, ICU session; Fol., follow-up session;

* *Bonferroni correction. The bold values indicate to significant results*.

### Physiological Parameters

Compared to the initial measurement at the beginning of the stimulation (first 15 s) and the last 15 s of the stimulation, the respiratory rate decreased significantly in all three sessions (Figure 3 and Table 4). The significant reduction in respiratory rate by using a directed hypothesis was −1.34 breath/min in the pre-ICU session, −0.56 breath/min in the ICU session and −1.88 breath/min in the follow-up session. Analysis of variance demonstrated no significant differences between the three sessions [*F*_(2, 84)_ = 0.97, *p* = 0.384]. No significant reduction or differences were observed during the three sessions in heart rate [*F*_(2, 86)_ = 0.49, *p* = 0.615] and blood pressure [*F*_(2, 77)_ = 0.22, *p* < 0.803]. Furthermore, the medication did not have any influence on the analysis.

**Table 4:** Respiratory rate during the three sessions.

**Variables**	**Pre-ICU session (breath/min)**	**ICU session (breath/min)**	**Follow-up session (breath/min)**
Mean Start (SD)	21.16 (4.68)	21.32 (3.57)	19.23 (4.91)
Mean End (SD)	19.82 (4,67)	20.76 (2.78)	17.35 (3.70)
Difference End Start (SD)	−1.34 (3.93)	−0.56 (1.79)	−1.88 (4.65)
*T*-test	*t*_(28)_ = −1.84, *p* = **0.039**	*t*_(29)_ = −1.72, *p* = **0.048**	*t*_(28)_ = −2.14, *p* = **0.021**

*SD, Standard Deviation. The bold values indicate to significant results*.

### Health-Related Quality of Life and Cognitive Functions

The health-related quality of life between the pre-ICU (M = 78.00, SD = 13.27) and follow-up sessions (M = 85.24, SD = 9.52) increased significantly by 7.03 (95% CI 1.26–12.81, *t*_(31)_ = 2.48, *p* < 0.019). No significant differences were found in cognitive functions between the pre-ICU (M = 27.52, SD = 2.01) and follow-up sessions (M = 27.56, SD = 2.02).

## Discussion

### Primary Results

Consistent with our first hypothesis, our results indicate that VR stimulation was pleasant, immersive, and easy to use. Furthermore, VR stimulation did not promote discomfort. Secondly, critically ill patients were able to perceive and process VR stimulation in the ICU session, whereas on a lower level compared to the pre-ICU and follow-up sessions. Thirdly, VR stimulation decreased the respiratory rate, which can be interpreted as a sign of relaxation, produced by isolating and protecting critically ill patients from the noisy and stressful environment.

### Acceptance, Comfort, and Recollection

Firstly, VR stimulation was highly appreciated and accepted by critically ill patients as reflected in high usability and satisfaction scores close to the maximum of the respective scales. Immersion was also highly scored, thus indicating that patients forgot about their actual surroundings. This may underline the fact that sensory overload and deprivation (stress and noise of the ICU environment) can be reduced during VR stimulation.

Furthermore, VR stimulation did not evoke any negative reactions, but provided patients with a confident, secure, fascinating and familiar environment. Almost 85% of all critically ill patients had clear memories of the VR stimulation during the ICU stay, as indicated by remembered content. However, only one third of the patients had clear memories of their stay in the ICU. The low recollection of the ICU stay was potentially caused by the psychological phenomenon of fading affect bias, which causes memories of negative events like the ICU stay to fade faster than the pleasant VR stimulation (34–36). These results and the low withdrawal rate of four patients further confirms that VR stimulation was highly appreciated, captured patients' attention and distracted them from the stressful and noisy ICU.

### Eye Movements

Visual fixation duration was significantly higher in the ICU session compared to pre-ICU and follow-up sessions. In contrast, the number of fixations was significantly lower in the ICU session, indicating that patients needed more time to process the information and had less visual exploration. This means that patients were experiencing post-surgery fatigue (i.e., reduced level of attention), potentially caused by medication (e.g., analgesics to reduce pain) and sleep-deprivation. Visual fixation duration and the number of fixations in the pre- and follow-up session were in line with VR stimulation in healthy volunteers (18) and findings when viewing dynamic natural scenes on a screen (37).

Additionally, post-surgery fatigue was also reflected in the number of gazed meaningful moving objects inside the video, which was significantly lower in the ICU session, but still at a high level of almost 40 objects a minute. It is noteworthy that there were no significant differences between the times fixating meaningful objects, confirming the second finding that patients were able to perceive, process, and follow the objects during all three sessions, although to a lesser extent during the ICU session. In line with the Attention Restoration Theory, the results suggest that critically ill patients are able to recover and restore attentional fatigue during VR nature stimulation by fostering low-effort attention (38, 39).

### Physiological Parameters

The third main finding was that the respiratory rate decreased significantly during all three sessions, whereas heart rate and blood pressure were not significantly influenced by the stimulation. Consistent with the Stress Recovery Theory, the VR stimulation had a relaxing effect on the parasympathetic nervous system. The decrease in respiratory rate during stimulation may indicate that nature-related VR stimulation can reduce stress and mental fatigue in critically ill patients by sealing off and protecting them from the noisy and stressful environment (14, 40).

One explanation why the significant relaxing effect was only seen in the respiratory rate was that more than 50% of all patients received medication affecting the heart rate and blood pressure, which influenced and thus stabilized and minimized the relaxing effect. Furthermore, the smaller decrease in the respiratory rate during the ICU session was probably due to medication influencing the respiratory rate, which was not administered in the pre-ICU and follow-up sessions.

### Health-Related Quality of Life and Cognitive Functions

In the follow-up session, health-related quality of life was significantly higher compared to the pre-ICU session, indicating that they profited from the surgery, whereas no significant differences were found for cognitive functions. Therefore, in line with the literature, the short stay in the ICU did not have a negative effect on long-term cognitive functions (4, 5).

### Limitations and Outlook

It remains unclear whether our findings can be generalized to a general population of critically ill patients. Moreover, as patients need to be awake, VR stimulation may not be suitable for critically ill patients who are unconscious and deeply sedated. In addition, it is unclear which part of the positive relaxing effect was due to the head-mounted display and which part due to the noise-canceling headphones. If the relaxing effect was due to noise reduction, VR stimulation might additionally help distract patients from their current condition and daily routine and thus has a positive effect on well-being. Therefore, the main limitations of this study design are the lack of a control group (i.e., sham intervention), the selected patient population (i.e., no patients were included in the study with prolonged ICU stay and mechanical ventilation) and the lack of repeated VR stimulation during ICU stay (i.e., long-term effects).

In this study, we showed that patients can be protected from the noisy and stressful environment. Further randomized clinical trials should investigate whether the proposed VR stimulation reduces functional and long-term cognitive impairment and improves health-related quality of life after ICU stay. Since long-term cognitive impairment is associated with delirium, studies should also focus on whether VR stimulation can potentially reduce the incidence and the duration of delirium (5). Furthermore, it remains unclear how long and how many times a day VR stimulation should be employed. We suggest including VR stimulation at least twice in the daily routine. If applicable, Electroencephalogram monitoring should be considered as a further means of measuring the effect of VR stimulation in critically ill patients.

## Conclusion

Our results indicate that the newly developed VR stimulation method was well accepted, immersive and appreciated by critically ill patients, and that VR was recollected better than the rest of the ICU stay. Furthermore, VR stimulation may have had a relaxing and calming effect, was easy to use and did not evoke discomfort. Eye movement analysis indicated that critically ill patients were able to perceive and process the stimulation in all sessions. Thus, VR stimulation has the potential of becoming a new method to reduce long-term cognitive impairment and comfort critically ill patients during a stay in ICU.

## Data Availability Statement

The datasets generated for this study are available on request to the corresponding author.

## Ethics Statement

The studies involving human participants were reviewed and approved by Ethics Committee of the Canton of Bern, Switzerland (KEK-Nr. 2016-01652). The patients/participants provided their written informed consent to participate in this study. Written informed consent was obtained from the individual(s) for the publication of any potentially identifiable images or data included in this article.

## Author Contributions

SG, M-MJ, UM, RM, SJ, and TN designed the study. SG developed the set-up and stimulated all critically ill patients together with M-MJ. SG and SK analyzed the data. SG, M-MJ, SK, UM, RM, SJ, and TN wrote the manuscript. All authors approved the final manuscript.

### Conflict of Interest

M-MJ and SJ report grants from Orion Pharma, Abbott Nutrition International, B. Braun Medical AG, CSEM AG, Edwards Lifesciences Services GmbH, Kenta Biotech Ltd, Maquet Critical Care AB, Omnicare Clinical Research AG, Nestle, Pierre Fabre Pharma AG, Pfizer, Bard Medica S.A., Abbott AG, Anandic Medical Systems, Pan Gas AG Healthcare, Bracco, Hamilton Medical AG, Fresenius Kabi, Getinge Group Maquet AG, Dräger AG, Teleflex Medical GmbH, Glaxo Smith Kline, Merck Sharp and Dohme AG, Eli Lilly and Company, Baxter, Astellas, Astra Zeneca, CSL Behring, Novartis, Covidien, and Nycomed outside the submitted work. The money was paid into departmental funds. No personal financial gain applied. The remaining authors declare that the research was conducted in the absence of any commercial or financial relationships that could be construed as a potential conflict of interest.
